# A Novel Approach to Estimating the Cortical Sources of Sleep Spindles Using Simultaneous EEG/MEG

**DOI:** 10.3389/fneur.2022.871166

**Published:** 2022-06-16

**Authors:** Dimitrios Mylonas, Martin Sjøgård, Zhaoyue Shi, Bryan Baxter, Matti Hämäläinen, Dara S. Manoach, Sheraz Khan

**Affiliations:** ^1^Department of Psychiatry, Massachusetts General Hospital and Harvard Medical School, Boston, MA, United States; ^2^Athinoula A. Martinos Center for Biomedical Imaging, Charlestown, MA, United States; ^3^Carle Illinois Advanced Imaging Center, Carle Foundation Hospital, Urbana, IL, United States; ^4^Department of Radiology, Massachusetts General Hospital and Harvard Medical School, Boston, MA, United States

**Keywords:** sleep spindles, MEG (magnetoencephalography), EEG, source localization, cortical sources, stage 2 NREM sleep, sleep oscillations

## Abstract

Sleep spindles, defining oscillations of stage II non-rapid eye movement sleep (N2), mediate sleep-dependent memory consolidation. Spindles are disrupted in several neurodevelopmental, neuropsychiatric, and neurodegenerative disorders characterized by cognitive impairment. Increasing spindles can improve memory suggesting spindles as a promising physiological target for the development of cognitive enhancing therapies. This effort would benefit from more comprehensive and spatially precise methods to characterize spindles. Spindles, as detected with electroencephalography (EEG), are often widespread across electrodes. Available evidence, however, suggests that they act locally to enhance cortical plasticity in the service of memory consolidation. Here, we present a novel method to enhance the spatial specificity of cortical source estimates of spindles using combined EEG and magnetoencephalography (MEG) data constrained to the cortex based on structural MRI. To illustrate this method, we used simultaneous EEG and MEG recordings from 25 healthy adults during a daytime nap. We first validated source space spindle detection using only EEG data by demonstrating strong temporal correspondence with sensor space EEG spindle detection (gold standard). We then demonstrated that spindle source estimates using EEG alone, MEG alone and combined EEG/MEG are stable across nap sessions. EEG detected more source space spindles than MEG and each modality detected non-overlapping spindles that had distinct cortical source distributions. Source space EEG was more sensitive to spindles in medial frontal and lateral prefrontal cortex, while MEG was more sensitive to spindles in somatosensory and motor cortices. By combining EEG and MEG data this method leverages the differential spatial sensitivities of the two modalities to obtain a more comprehensive and spatially specific source estimation of spindles than possible with either modality alone.

## Introduction

Sleep spindles, a defining oscillation of stage II non-rapid eye movement sleep (N2), are brief (~1 s) powerful bursts of 12–15 Hz activity initiated in the thalamic reticular nucleus (TRN) (1, 2) and propagated to the cortex *via* thalamocortical circuitry (3). Sleep spindles are typically separated based on their frequency into slow (9–12 Hz) and fast spindles [12–15 Hz; (4, 5)]. Although both spindle classes are generated in TRN they have different cortical topographies with slow spindles being more prominent at frontal and fast spindles at central and parietal electrodes (6, 7). In humans sleep spindles correlate with sleep-dependent memory consolidation, learning efficiency, and IQ [for a review see (8)]. Sleep spindles are disrupted in several neurodevelopmental, neuropsychiatric, and neurodegenerative disorders characterized by cognitive impairment (9). Importantly, increasing spindles both pharmacologically (10–12) and using non-invasive brain stimulation (13) can improve memory, consistent with evidence from optogenetic studies of rodents indicating a causal role in memory consolidation (14, 15). This provides an impetus to target spindles to treat cognitive deficits (16). Since spindles act locally to mediate memory typically in regions involved in initial learning (17–20), this effort would benefit from a more spatially precise measurement of spindles. In humans, spindles are typically detected with EEG. Relatively few studies have used magnetoencephalography (MEG) to complement EEG spindle detection (21–30). Here we describe a new method using simultaneously acquired EEG and MEG data from afternoon naps to comprehensively characterize sleep spindles and to estimate their cortical sources.

Compared with EEG, MEG is more sensitive to focal cortical spindle sources but mainly detects sources that are tangential to the cortical surface (31, 32). In contrast, EEG detects both radial and tangential sources. Spindles detected only by MEG sensors tended to be more focal and did not propagate across the cortex, whereas spindles detected in both modalities were first detected by MEG and then detected by EEG after spreading to additional regions (23). These studies suggest that (i) MEG is more sensitive to the emergence of non-synchronous bursts of focal spindles due to its more confined spatial sensitivity; (ii) EEG is more likely to detect spindles that cover extended areas on the cortex, and (iii) because of their complementarity, MEG and EEG together provide more accurate source estimation than either technique alone (33). A more spatially specific estimation of sleep spindle sources is important given the role of local spindles in mediating memory (17–20).

Here, we present a novel method to estimate the cortical sources of spindles using simultaneous EEG/MEG recordings, constrained to the cortex based on structural MRIs, during an afternoon nap. To validate this method, we compared spindles detected in source space to those detected on the scalp (sensor space) using EEG (gold standard). We next evaluated the spatial distribution of spindles that were common and unique to each modality by comparing source space spindle detection using EEG only, MEG only and combined EEG/MEG. We conclude by discussing the advantages of using combined EEG/MEG for detecting and source localizing spindles over either technique alone.

## Materials and Methods

### Participants

Thirty one healthy adults were recruited from the community through advertisements and were screened to exclude a history of mental illness diagnosed sleep disorders, treatment with sleep medications, pregnancy, and a history of head injury, neurological disorder and substance abuse or dependence within the past 6 months. All participants gave written informed consent and were paid for participation. The study was approved by the Partners Human Research Committee. Participants were asked not to consume caffeine or alcohol on the day of the recording. All 25 participants (age 29 ± 6, 21–42; 19 males) who produced valid nap data (>10 min of artifact rejected N2 sleep) were included.

### Procedure

All participants completed two visits at least 1 week apart. The first visit (Nap 1) acclimated the participant to napping in the MEG scanner and was followed by a second visit (Nap 2). Participants were wired for polysomnography (PSG) and given a 90 min afternoon nap opportunity with simultaneous EEG and MEG recording while lying supine in the MEG scanner. Before the nap we recorded 5 min of quiet rest during which participants were instructed to maintain fixation on a cross in the center of the screen. After their second visit participants returned for an MRI scan.

### EEG/MEG Data Acquisition

Data were recorded using a 306 channels whole-head Elekta-Neuromag MEG system [Elekta Oy (now MEGIN, Croton Healthcare), Helsinki, Finland] in a magnetically shielded room (IMEDCO, Hagendorf, Switzerland) simultaneously with 70 channels of EEG, submental electromyography (EMG) and 2 electrooculography electrodes (EOG). All signals were digitized at 600 Hz. The MEG sensors are arranged as triplets at 102 locations; each location contains one magnetometer and two orthogonal planar gradiometers. Locations of the EEG electrodes and ~200 head shape points were recorded using a 3D digitizer (Polhemus FastTrack). Four head position index (HPI) coils were used to continuously track the position of the head relative to the scanner.

### EEG/MEG Data Pre-processing

We applied the signal space separation (SSS) algorithm (34) to the MEG signals to suppress environmental noise and correct for head movements using the HPI coils. Sleep data were low-pass filtered at 60 Hz and down-sampled to 200 Hz using MNE software for further analysis (35). Each 30 s epoch of EEG data was visually scored according to standard criteria as WAKE, REM, N1, N2, or N3 (36) by expert raters (Table 1). Sleep quality was quantified using sleep onset latency (SOL), total sleep time (TST), time in bed (TIB), sleep efficiency (TST/TIB), and wake after sleep onset (WASO).

**Table 1:** Means, standard deviations of participants' sleep quality, and architecture measures.

	***Mean*** **±*sd (min–max)***
**Sleep quality**	
^*^TIB	92 ± 5 (80–105) min
^*^TST	65 ± 23 (19–96) min
^*^SOL	6 ± 7 (1–26) min
^*^WASO	21 ± 19 (1–65) min
Sleep efficiency	70 ± 24 (21–98)%
**Sleep architecture**	
N1	14 ± 6 (4–23) min
N2	38 ± 19 (11–76) min
N3	10 ± 12 (0–37) min
REM	3 ± 6 (0–20) min

* *TIB, Time in bed; TST, Total sleep time; SOL, Sleep onset latency; WASO, Wake after sleep onset*.

EEG and MEG data were pre-processed and analyzed using custom scripts in MATLAB (MathWorks, Natick MA), FieldTrip (37) and MNE software (35). Sleep data were band-pass filtered at 0.3–35 Hz and electrodes displaying significant artifacts were spatially interpolated. EEG data were then re-referenced to the common average. Resting state data were notch-filtered at 60 Hz. Signal space projection [SSP; (38)] implemented in MNE was used to remove cardiac artifacts, and remaining artifacts were visually identified and removed. Artifact-free data from N2 sleep were used for further analyses. Although spindles also occur during N3 sleep, we restricted our analyses to N2 since spindle physiology differs across sleep stages and only 8 of 25 participants had more than 10 min of N3.

### MRI Acquisition

Anatomical images were acquired on a 3T Siemens Trio whole-body MRI system (Siemens Medical Systems, Erlangen, Germany) with a 32-channel head coil. The images were acquired using a 3D RF-spoiled magnetization prepared rapid gradient echo (MP-RAGE) sequence (TR = 2,530 ms; TE = 1.7/3.6/5.5/7.3 ms; Flip Angle = 7°; FOV = 256 mm, 176 in-plane sagittal 1 mm isotropic slices, scan duration 6 m 12 s). In addition, a multi echo flip angle (5°) FLASH pulse sequence was employed to obtain data for constructing individual boundary element model (BEM) surfaces for forward modeling (610 Hz per pixel, TR = 20 ms, TE = 1.89 + 2 n ms (*n* = 0–7), 128 in-plane sagittal slices sized 1 × 1.33 mm, 1.33 mm thickness).

### Source Reconstruction

Co-registration of the EEG and MEG sensors to each participant's structural MRI was implemented in MNE using the digitized electrodes, fiducials, HPI coils and head shape points. MRI reconstruction and tissue segmentation were performed using FreeSurfer (39, 40). The FreeSurfer-derived cortical surface tessellation was decimated to a regular source dipole grid with 3 mm spacing between adjacent source locations, corresponding to ~18,500 dipoles. The forward solutions were then computed using the three-layer BEM (41) using inner, outer skull, and scalp surfaces from segmentations of the FLASH images.

**Figure 1:**
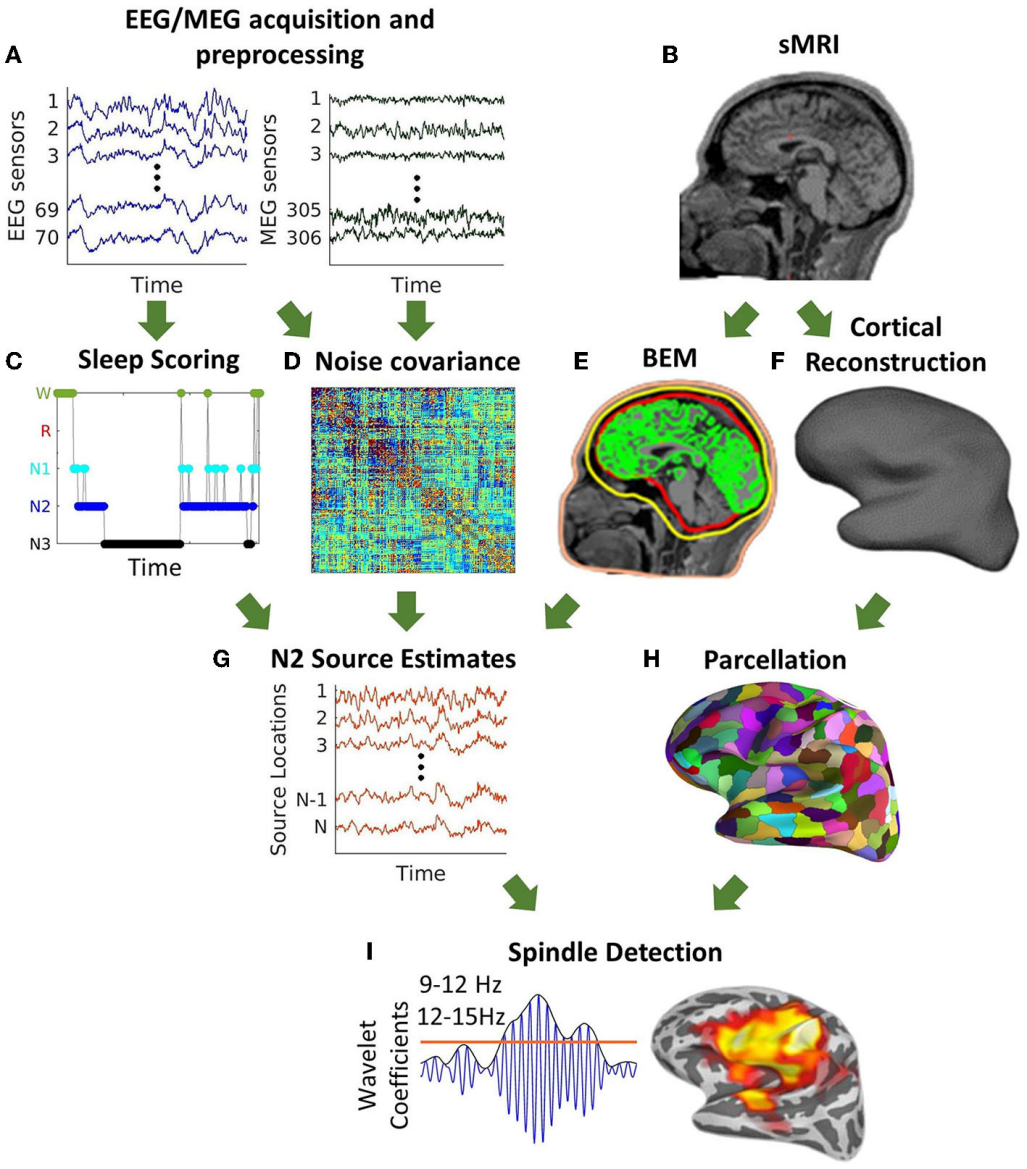
Schematic description of source space spindle detection. **(A)** Pre-processing of simultaneously acquired EEG/MEG data. **(B)** Structural MRI. **(C)** Sleep scoring of nap data. **(D)** Noise covariance estimates calculated using the EEG/MEG data from the 5 min resting-state scan filtered at 100–140 Hz. **(E)** Construction of a three-layer boundary element model (BEM) surfaces (inner, outer skull, and scalp) for forward modeling **(F)** Cortical reconstruction. **(G)** Source estimates of N2 calculated using the cortically constrained minimum-norm estimate of cortical currents. **(H)** Parcellation of the cortical surface into 448 regions. **(I)** Automatic spindle detection at each cortical region using a wavelet-based detector.

The cortically constrained minimum-norm estimate of the cortical currents [MNE; (42, 43)] was computed with source orientations fixed perpendicular to the local cortical surface and a regularization factor of 0.1. Noise covariance estimates were calculated using data from the 5 min resting-state scan filtered at 100–140 Hz. We used dynamical statistical parametric mapping [dSPM; (44)] to reduce the MNE inverse solution bias toward superficial cortical sources. FreeSurfer was used to automatically parcellate the cortex into 72 regions (45). After discarding “medial wall" and “corpus callosum,” these regions were further parcellated into a total of *N* = 448 similarly sized cortical regions using FreeSurfer (46). The resulting source-space time courses of artifact-free N2 sleep were then computed in these 448 regions. In order to align the signs of the time series across dipoles within a label, we used the singular value decomposition (SVD) of the data. The sign of the dot product between the first left singular vector and all other time-series in a label was computed. If this sign was negative, we inverted the time-series before averaging. The same procedure was followed to generate three source localization estimates, from EEG alone, MEG alone, and combined EEG/MEG data. For analytic methods overview see Figure 1.

### Spindle Detection

Slow and fast spindles were automatically detected in the 9–12 and 12–15 Hz band-pass-filtered data respectively, at each sensor and cortical region using a wavelet-based algorithm (47, 48). Specifically, based on temporally smoothed (window duration = 0.1 s) wavelet coefficients (from a complex Morlet wavelet transform), spindles were identified as intervals exceeding 9 times the median for at least 400 ms. The frequency range for spindle detection, defined based on the full-width half-maximum of the wavelet amplitude response in the frequency domain (49), was chosen based on prior studies and to minimize the overlap between the two spindle classes (Supplementary Figure 1) (4, 47, 48). The threshold for spindle detection was chosen to maximize the between class (“spindle” vs. “non-spindle”) variance (50) based on data from healthy participants in a previous study (47). This detector has been validated against visual inspection in healthy people, individuals with schizophrenia and children with autism spectrum disorder (47, 51). The duration of individual spindles was measured in 2 s epochs centered on the point of spindle detection as the full width half max of the wavelet energy.

### Definition of Spindle Events in Sensor and Source Space

**Figure 2:**
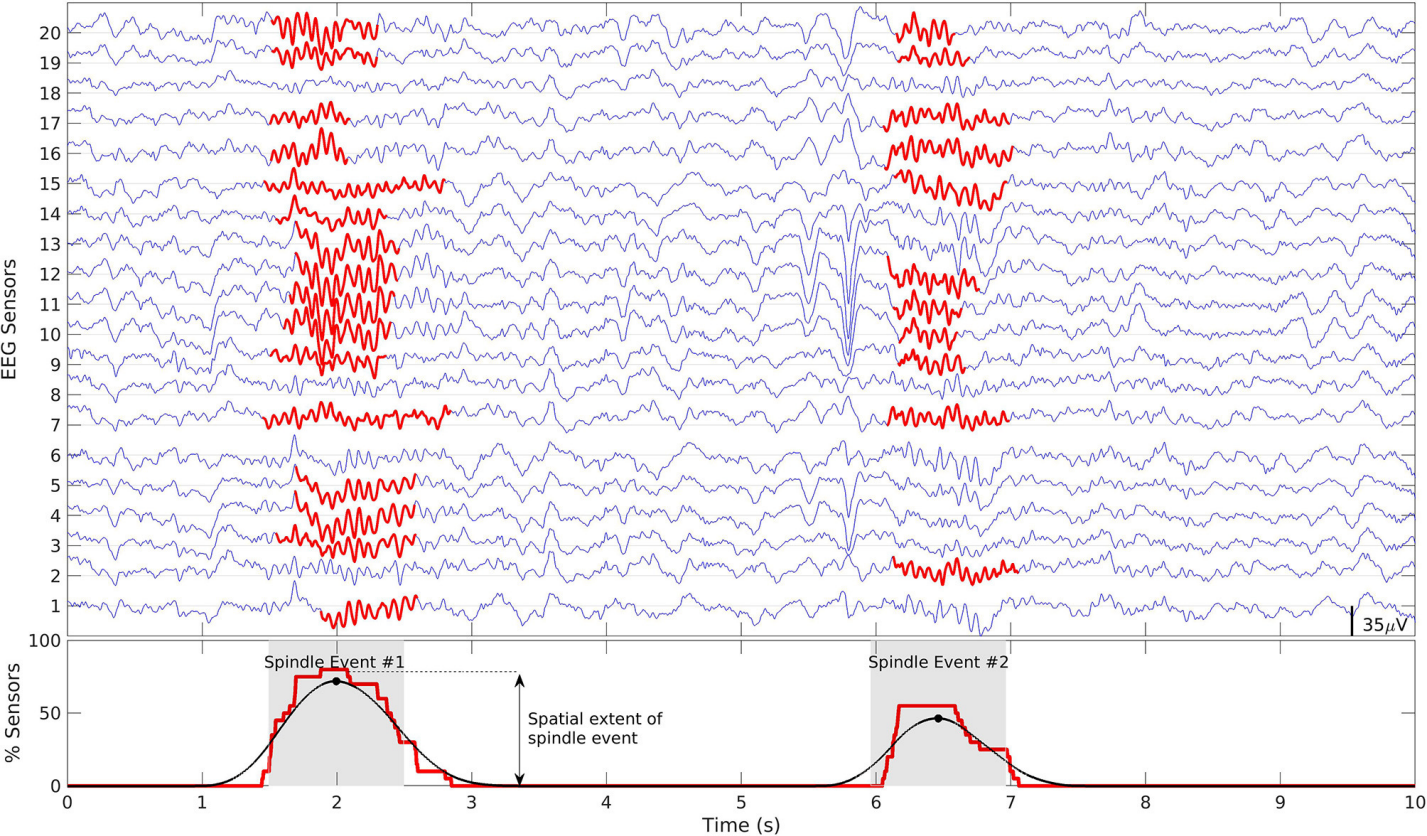
Definition of spindle events. Top: Example of 10 s of N2 signal from 20 EEG electrodes. Detected spindles at each sensor are highlighted in red. Bottom: The raw aggregate signal (red) and smoothed signal (black). Spindle events were defined as 1 s time-windows around the peaks of the smoothed signal (gray patch). The maximum amplitude within this window reflects the spatial extent of the detected spindle event. The same definition applies to MEG sensors and source space analyses.

As there is no one-to-one correspondence between scalp sensors and source space regions we defined windows of spindle activity in both so that we could compare spindle detection in each. To define windows of spindle activity we first assigned a binary value (*y*_*i*_) to each sensor/region at each time point that was set to one if a spindle was detected and zero if not. The binary signals were summed across all sensors/regions resulting in an aggregate signal (*Y*) which was >0 when a spindle was detected at any of the sensors/regions at any given time-point. After smoothing *Y* with a 500 ms moving average, we detected the temporal local maxima using the MATLAB function *findpeaks*. To avoid detection of spurious spindle activity a minimum distance between maxima was set at 500 ms and a minimum extent was set at 1% of sensors/regions. One second windows centered at the detected local maxima were defined as temporal windows of spindle activity across sensors/regions (Figure 2). We will refer to these periods of spindle activity across sensors/regions as “spindle events” to distinguish them from spindles detected at each sensor/region (e.g., see Supplementary Figures 1, 4). The duration of the windows was set at 1 s. The spatial extent of a spindle event (i.e., the total number of sensors/regions where a spindle was detected) was quantified as the maximum amplitude of *Y* (Figure 2). To account for different sleep durations, we calculated spindle event density (i.e., spindle events per minute). In contrast to the typical definition of spindle density at each sensor/region, spindle event density is based on the definition of spindle events across multiple sensors/regions. Using this method, we first compared spindle events from sensor vs. source space EEG to validate spindle detection in source space. We then compared the density and spatial extent of source space spindle events detected in EEG alone, MEG alone and combined EEG/MEG data.

### Validation of Spindle Detection

We first validated spindle detection in source space by quantifying the correspondence of source space EEG estimates with scalp EEG (i) between subjects, by correlating the total number of spindle events in source vs. sensor space and (ii) within subjects, by calculating the temporal overlap using the *F1* score of detected spindle events in source vs. sensor space. We defined temporal overlap as ≥20% [*F1* scores for different overlap values (10–50%) and window lengths (0.4–2 s) are presented in Supplementary Figure 2]. Spindle event density in source vs. sensor space was compared using a paired *t*-test.

To calculate F1 scores we defined (i) false positives (*FPs*), as spindle events detected in source but not sensor space, (ii) false negatives (*FNs*) as spindle events detected in sensor but not source space, and (iii) true positives (*TPs*) as spindle events detected in both sensor and source space. Precision (*f*_P_), recall (*f*_R_), and the *F1* score were calculated as follows:


$$\begin{array}{lll} f_{P} & = & TP/\left(TP+FP\right);\\ f_{R} & = & TP/\left(TP+FN\right);\\ F1\; & = & \frac{2f_{P}f_{R}}{f_{P}f_{R}}. \end{array}$$

To evaluate whether the spatial extents of spindle events detected in sensor and source space (i.e., *TPs*) were related, we correlated *Y* in the sensor space (the number of sensors showing that spindle) with *Y* in the source space (number of regions).

Since spindle detection in source space was more prone to *FPs* than *FNs* (see Results), we asked where these *FPs* were more likely to be detected, by calculating the percent of *FPs* detected at each region. We then tested whether these source-detected “*FPs”* might actually reflect sub-threshold sensor space spindle activity. For each *FP* spindle event detected in the source space we calculated the sigma power across all EEG sensors using the squared amplitude of the Hilbert transform, after bandpass filtering at 9–12 Hz for slow and 12–15 Hz for fast spindles. We then z-normalized it against the power of randomly selected spindle-free 1 s periods and averaged across time-points and sensors.

## Test-Retest Reliability of Spindle Events

Previous studies have demonstrated that sleep spindles are a heritable trait-like feature of the scalp EEG (5, 52) and are stable within individuals across nights and naps (48, 53). Here we wanted to investigate whether this is also true for spindle events detected in source space using different modalities. We calculated intraclass correlation coefficients (ICCs) for scalp EEG, source EEG, MEG, and EEG/MEG in the 19 subjects who had valid data from two naps. To calculate ICCs, we estimated between- and within-subjects variances of spindle event density from regression models with subject as a random effect. To compare the reliability of spindle event density among modalities we estimated the 95% confidence intervals (CIs) of the ICCs based on 1,000 bootstrap samples.

### Comparison of EEG Alone, MEG Alone, and Combined EEG/MEG Detected Spindle Events in Source Space

To determine whether source-space EEG, MEG, and EEG/MEG are differentially sensitive to spindles we compared the spindle events detected by each modality. The density of spindle events was compared with a linear mixed effects model with Modality as a fixed effect (EEG, MEG, and EEG/MEG) and Subject as a random effect. The correspondence of spindle events detected by EEG, MEG, and EEG/MEG, based on their temporal overlap, was calculated using the same method as above. To compare the spatial specificity of the source estimates we first used pairwise comparisons of the spatial extent of spindle events detected by EEG, MEG, and EEG/MEG. We then examined the spatial extent and topography of spindle events detected with only one of two estimates (e.g., spindle events unique to EEG only). To investigate whether there were cortical regions at which one modality was more sensitive to spindle events, for each region we calculated the percent of spindle events that were detected by only one of two estimates.

## Results

We focus on fast spindles (defined as 12–15 Hz), which are a well-replicated biomarker of overnight memory consolidation (8) and disrupted in neuropsychiatric disorders, particularly schizophrenia (9). Slow spindle (defined as 9–12 Hz) findings are described in Supplementary Results and Supplementary Figures 1, 3, 5–9.

### Validation of EEG Source Space Spindle Detection

**Figure 3:**
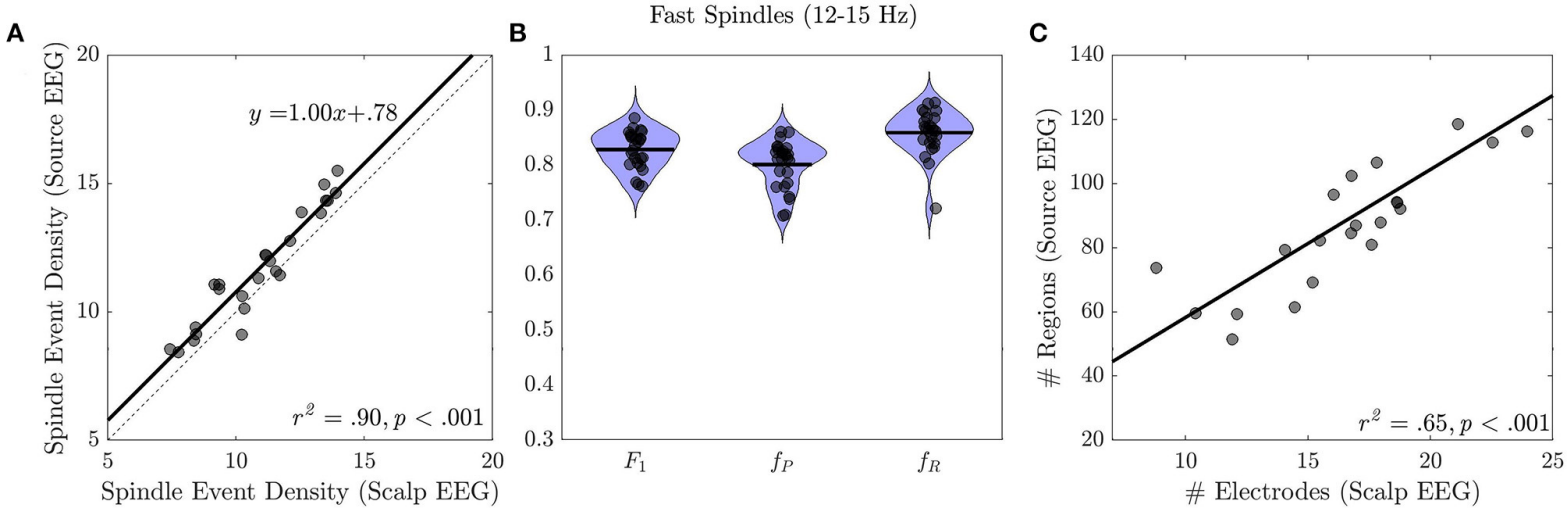
Spindle events in source vs. sensor space EEG. **(A)** Spindle event density in source vs. sensor space. Regression line (black solid) and the identity line (gray dashed) are shown. **(B)** Correspondence of spindle events detected at the source vs. the sensor space (F1 = 0.83 ± 0.03, fP = 0.80 ± 0.04, fR = 0.86 ± 0.04). **(C)** Spatial extent of spindle events in source vs. sensor space with regression line.

Source and sensor space EEG spindle event density were highly correlated (*r*^2^ = 0.90, *p* < 0.001, slope=1.00 ± 0.07, Intercept: 0.78 ± 0.78; Figure 3A). On average EEG spindle event density was 7% higher in source space than in sensor space (sensor space: 10.92 ± 2.04; source space: 11.70 ± 2.15; *t* = 5.72, *p* < 0.001). In within-subjects analyses, 86% of spindle events detected in sensor space temporally overlapped with spindle events detected in source space, while 80% of spindle events detected in source space overlapped with spindle events measured in sensor space (*F1* = 0.83 ± 0.03, *f*_P_ = 0.80 ± 0.04, *f*_R_ = 0.86 ± 0.04; Figure 3B). Spindle events on the scalp were detected on average at 18/70 (26%) sensors and at 73/448 (21%) cortical regions in source space. The spatial extent of spindle events in source and sensor space was highly correlated (*r*^2^ = 0.65, *p* < 0.001; Figure 3C).

**Figure 4:**
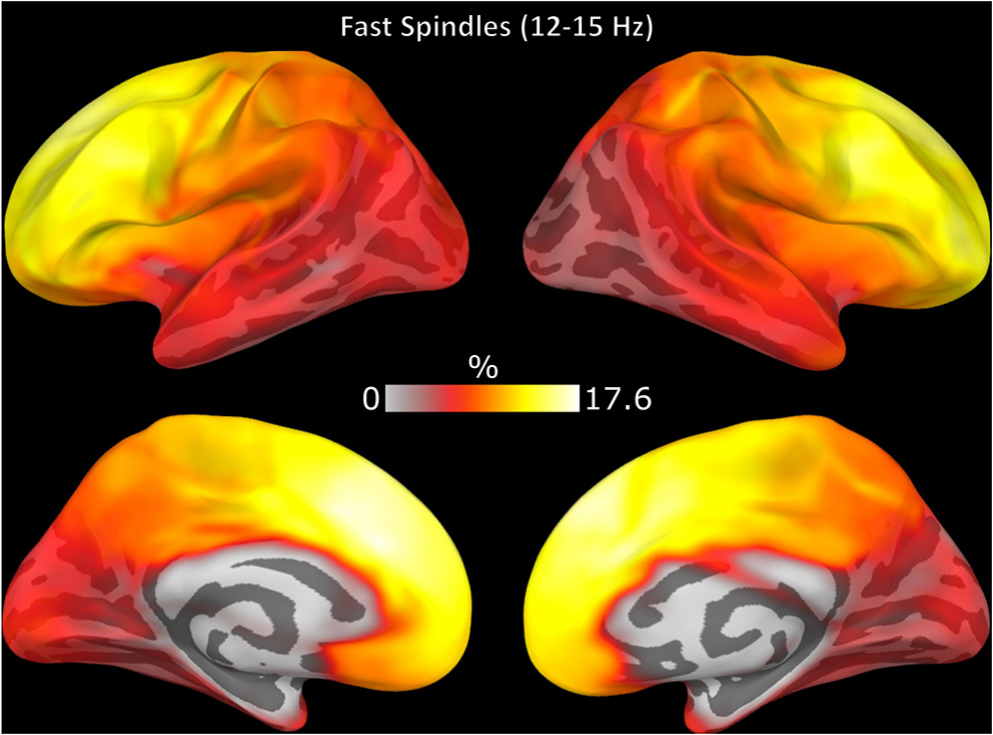
Topography of spindle events detected only in source space EEG (FPs). The color of each region represents the number of FPs expressed in this region over the total number of FPs as a percentage.

Spindle events that were detected in source but not sensor space (*FPs*) was more likely to be detected in frontal cortex (Figure 4). On average, for each subject 36% (range 24–51%) of spindles detected only in source space (*FPs*) had significantly elevated sigma power in sensor space (*z* > 1.69) suggesting that these *FPs* might reflect sub-threshold spindle activity.

### Test-Retest Reliability of Spindle Events

**Figure 5:**
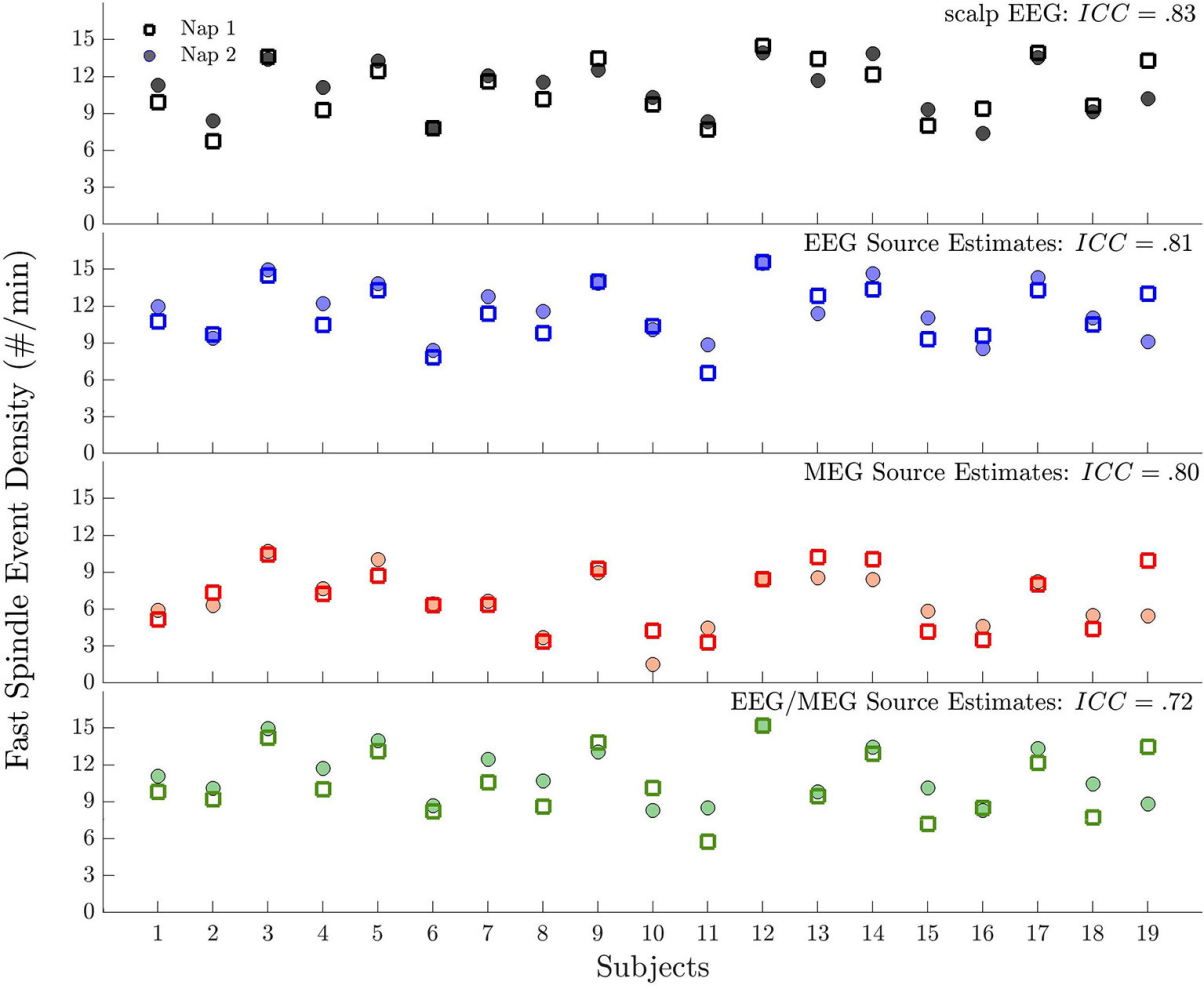
Test-retest reliability of spindle events across naps for each modality. Plot of spindle event density for each subject during Nap 1 and Nap 2. Spindle events were detected (from top to bottom) at scalp EEG, source EEG, MEG, and EEG/MEG.

As in previous studies (5, 48, 52, 53) sensor space EEG spindle events were stable within individuals across two naps [ICC = 0.83, CI: (0.66, 0.91)]. Similarly, source space detected spindle events were stable across naps and ICCs did not differ significantly [i.e., their CIs overlapped; EEG: ICC = 0.81, CI: (0.55, 0.92); MEG: ICC = 0.80, CI: (0.56, 0.93]; EEG/MEG: ICC = 0.72, CI: (0.42, 0.88); Figure 5].

### Comparison of Source Space Spindle Events Detected With EEG Alone, MEG Alone, and Combined EEG/MEG

**Figure 6:**
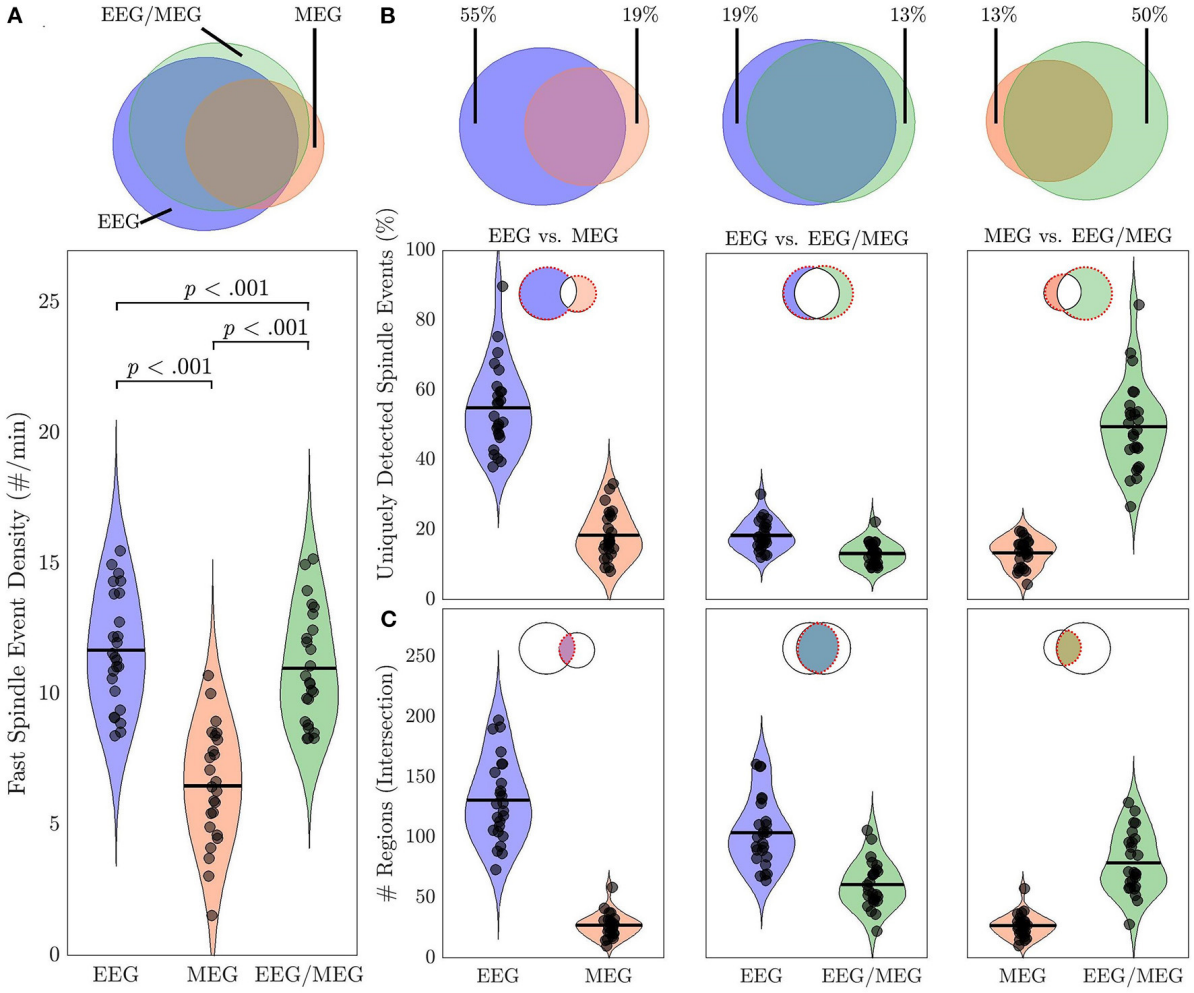
Spindle events in source space using EEG alone, MEG alone and combined EEG/MEG. **(A)** Venn diagram and violin plots depicting spindle event density in EEG, MEG, and EEG/MEG with *p*-values for pairwise comparisons. **(B)** Percent of uniquely detected spindle events by each modality for EEG vs. MEG, EEG vs. EEG/MEG, and MEG vs. EEG/MEG. **(C)** Spatial specificity of commonly detected spindle events (intersection of Venn diagrams). Spatial extent of spindle events detected by EEG and MEG, EEG and EEG/MEG, and MEG and EEG/MEG. Black circles represent individual data.

Overall spindle event density differed significantly between source estimates [*F*_(2, 72)_ = 238.46, *p* < 0.001): Spindle event density was lower in MEG than either EEG (44%; *t* = 15.78, *p* < 0.001) or EEG/MEG (40%; *t* = 16.61, *p* < 0.001), and lower for EEG/MEG than EEG (6%, *t* = 5.12, *p* < 0.001; Figure 6A). We excluded the possibility that this result was simply due to a higher absolute spindle detection threshold in MEG by showing that the threshold was higher in EEG (EEG: 2.91 ± 1.86, MEG: 0.62 ± 0.35; Wilcoxon *z* = 5.65, *p* < 0.001). Fifty-five percent of EEG-detected spindle events had no corresponding event in MEG. Conversely 19% of MEG-detected spindle events lacked a corresponding EEG event (Figure 6B). This indicates that although EEG detects more spindle events than MEG, the two modalities also detect different events. The combined EEG/MEG estimate captured more of the spindles present in EEG alone than MEG alone (*t* = 18.74, *p* < 0.001). Similarly, there were more common spindles between the combined EEG/MEG and the MEG alone than between the EEG alone and MEG alone (*t* = 5.95, *p* < 0.001). These data indicate that the combination of EEG and MEG provides a more comprehensive account of spindles than either modality alone.

The spatial distribution of spindle density differed across modalities with EEG showing maximum spindle density in lateral and medial frontal cortex extending into posterior cingulate cortex, while MEG spindle density was relatively low over prefrontal cortex and peaked in posterior cingulate cortex (Supplementary Figure 3).

**Figure 7:**
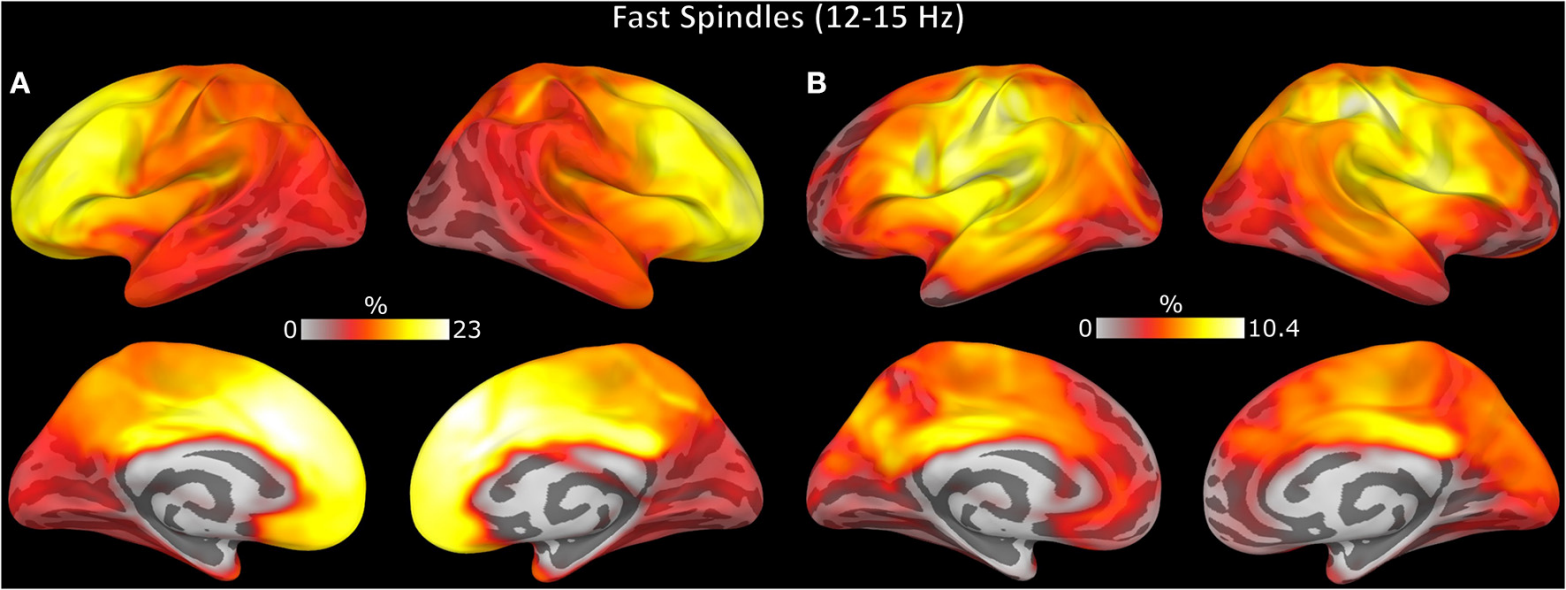
Topography of spindle events uniquely detected by **(A)** EEG and **(B)** MEG. The color represents the percent of spindle events detected in each region relative to the total spindle events detected.

Spindle events detected by EEG included more regions than MEG (*t* = 16.00, *p* < 0.001) or combined EEG/MEG (Figure 6C; *t* = 14.24, *p* < 0.001). EEG/MEG detected spindle events were more widespread than those detected by MEG (*t* = 15.70, *p* < 0.001; Figure 6C). More focal spindle events were less likely to be detected regardless of modality and, contrary to expectations, MEG was not more sensitive to focal events (Supplementary Figure 4). However, there were topographical differences between modalities: MEG was less likely than EEG to detect medial and lateral frontal spindle events (Figure 7A) and EEG was less likely than MEG to detect spindle events in motor and somatosensory cortex (Figure 7B).

## Discussion

We employed a novel method using simultaneous EEG and MEG recordings during sleep to estimate the cortical sources of sleep spindles. We first validated source space spindle detection with EEG by demonstrating strong agreement with sensor space (i.e., scalp) EEG spindle detection. We also extended previous findings that sensor space EEG spindles are stable across sessions to source space spindle estimates using EEG, MEG, and EEG/MEG. Finally, we show that by combining EEG/MEG data, anatomically constrained by structural MRI, we leverage the differential sensitivities of the two modalities to cortical sources to obtain a more comprehensive view of spindles and increase the spatial specificity of the source estimation compared to EEG or MEG alone.

The density of EEG spindle events detected in source space showed a good correspondence with those detected in sensor space, but on average was 7% higher for fast spindles and 19% higher for slow spindles. The significantly higher agreement between sensor and source space for fast spindles may reflect the reduced amplitude and increased variability of EEG slow spindle spectral peaks (4). During over a third of the spindle events detected in source but not sensor space, the averaged sigma power of scalp EEG electrodes was elevated suggesting that source detection was more sensitive to sub-threshold scalp EEG spindle activity. This may reflect that each EEG scalp electrode captures activity from multiple brain regions while the point spread functions in source space are more focal (31, 32, 54). Some of the remaining two thirds of spindles detected in source but not sensor space (i.e., “false positives”) may be more focal spindles whose signal is obscured by averaging across all electrodes or they may be noise.

We replicated previous findings that spindle activity is stable over sessions within individuals and extended these results to spindle events detected in source space regardless of modality. This is consistent with evidence that spindle activity is a heritable trait-like feature of the sleep EEG (5, 48, 52, 53). Although this was a nap study, spindle density during naps is a reliable estimate of overnight spindle density indicating that our findings can generalize to overnight sleep (55). Spindle event density using MEG and combined EEG/MEG source estimates was more variable within subjects compared to EEG, particularly for slow spindles. The increased number of MEG sensors compared to EEG (70 EEG vs. 306 MEG sensors) could potentially increase the variability of the measurements across sessions. Another possible explanation could be that although we track the head position and take any head motion into account in the post-processing (56, 57), different head positions across sessions could still affect the source estimates of MEG alone and EEG/MEG.

Spindle events detected exclusively by EEG or MEG had distinct topographical distributions. EEG was more sensitive to spindles in medial and lateral frontal cortex, while MEG was more sensitive to spindles in somatosensory and motor regions. These topographies may reflect differential sensitivity of EEG and MEG to spindles arising from two thalamocortical pathways: the core pathway that projects to middle cortical layers, particularly in somatosensory and motor regions, and the matrix pathway that projects diffusely to more superficial cortical layers (58, 59). Our findings support the hypothesis that EEG is more sensitive to widely expressed matrix spindles whereas MEG is more sensitive to focal core spindles (29, 60). The differential sensitivity of EEG and MEG may reflect that widely distributed sources lead to greater signal loss in MEG due to cancellation (61).

Contrary to a prior report (19), MEG detected significantly fewer spindle events than EEG. This may reflect MEG's relative insensitivity to radially oriented and distributed sources of some spindle activity. Our results are consistent with older studies that report more spindles detected with EEG than MEG (21, 27). The inconsistent results could reflect different methodology. Dehghani et al. (23) detected spindles using a spectral peak algorithm across EEG and MEG sensors during 2 min of N2 sleep whereas in this study we detected spindles on a sensor/region basis during all of N2 (mean duration: 38 min).

Spindles are generated in the thalamic reticular nucleus (1, 2) and are propagated to the cortex *via* thalamocortical circuitry (3). Since the contribution of subcortical sources to EEG is weak and to MEG even weaker, we restricted spindle detection to the cortical surface (62, 63). The lack of access to thalamic activity renders the question of what constitutes “true spindle activity” impossible to answer. Here we used spindle activity detected at the scalp EEG as the “gold standard,” to validate our spindle detection method in the source space. The lack of ground truth precludes any statements of which source estimate of spindles is the most valid. More sophisticated methods are needed to non-invasively assess the interaction between cortex and thalamus during spindle activity (63–65). Because this was an afternoon nap study, fewer than a third of the participants had more than 10 min of N3 sleep, not allowing us to investigate whether our findings generalize to N3.

Fast spindles mediate sleep-dependent memory consolidation, are disrupted in a number of neurodevelopmental and neurodegenerative disorders [for a review see (9)] and have been identified as a mechanistic biomarker of cognitive dysfunction and a potential treatment target, [e.g., see (16)]. Although spindles can be expressed widely in the cortex, they act in a spatially specific manner to induce the plasticity underlying memory consolidation. For example, during the sleep that follows training on a motor task, increased spindles and sigma power in the contralateral motor cortex correlates with improved performance upon awakening (17–20). In schizophrenia, spindle deficits correlate with both memory deficits and increased connectivity of the thalamus specifically with somatosensory and motor cortex (47, 66). Children with Rolandic epilepsy have a focal spindle deficit in the affected regions that correlates with cognitive and motor dysfunction (67). The spatial specificity that characterizes both the functionality of spindles in health, and their disruption in disorders highlights the utility of techniques with high spatial resolution for both basic and clinical studies of sleep-dependent cognition.

In summary we present a novel method that leverages the differential sensitivities of EEG and MEG to reveal the cortical sources of spindles. Combined EEG and MEG provide a more comprehensive detection and focal source estimation than either technique alone. Accurate estimation of spindle activity will illuminate the function of spindles, how it goes awry in disorders, and guide the development of more targeted treatments.

## Data Availability Statement

The raw data supporting the conclusions of this article will be made available by the authors, without undue reservation.

## Ethics Statement

The studies involving human participants were reviewed and approved by Partners Human Research Committee. The patients/participants provided their written informed consent to participate in this study.

## Author Contributions

DSM and MH contributed to conception and design of the study and contributed to all aspects of its completion. DM, MS, ZS, BB, and SK contributed to conceptualization, methods development, data analysis, and writing. All authors reviewed and commented on the finished product and contributed to the article and approved the submitted version.

## Funding

This work was supported by a grant from the Simons Foundation to the Simons Center for the Social Brain at MIT, K24 MH099421 and R01 MH092638 to DSM; 5P41EB030006 P41 Center for Mesoscale Mapping (NIBIB), 5U01EB023820 Device-Independent Acquisition and Real Time Spatiotemporal Analysis of Clinical Electrophysiology Data (NINDS) and 5R01NS104585 to MH; McKnight Clinical Translational Research Scholarship in Cognitive Aging and Age-Related Memory Loss, funded by the McKnight Brain Research Foundation through the American Brain Foundation and the American Academy of Neurology Institute to BB.

## Conflict of Interest

The authors declare that the research was conducted in the absence of any commercial or financial relationships that could be construed as a potential conflict of interest.

## Publisher's Note

All claims expressed in this article are solely those of the authors and do not necessarily represent those of their affiliated organizations, or those of the publisher, the editors and the reviewers. Any product that may be evaluated in this article, or claim that may be made by its manufacturer, is not guaranteed or endorsed by the publisher.
