# Supplementary material for: A Novel Approach to Estimating the Cortical Sources of Sleep Spindles Using Simultaneous EEG/MEG
Source: Front Neurol. 2022 Jun 16;13:871166. doi: 10.3389/fneur.2022.871166 (PMC9243385; doi:10.3389/fneur.2022.871166)
Supplement: Supplementary file 1 [file Data_Sheet_1.docx]

**Supplementary Material**

**Supplementary Results**

Validation of EEG source space slow spindle detection.

Source and sensor space EEG slow spindle event density were highly correlated (*r^2^*=.55, *p*=.001, slope=.70±.13, Intercept: 3.58±.97; SFigure 5a). On average EEG slow spindle event density was 19% higher in source space than in sensor space (sensor space: 7.26±1.50; source space: 8.65±1.41; *t*=6.64, *p*<.001). In within-subjects analyses, 80% of slow spindle events detected in sensor space temporally overlapped with slow spindle events detected in source space, while 67% of slow spindle events detected in source space overlapped with spindle events measured in sensor space (F1 = .73±.06, *f*_P_= .67±.09, *f*_R_=.80±.05; SFigure 5b). Spindle events on the scalp were detected on average at 10/70 (14%) sensors and at 52/448 (12%) cortical regions in source space. The spatial extent of spindle events in source and sensor space was highly correlated (*r*^2^=.79, p<.001; SFigure 5c).

Similar to fast spindle events, slow spindle events that were detected in source but not sensor space was more likely to be expressed in frontal cortex (Figure S6). On average, for each subject 34% (range 18%-59%) of spindles detected only in source space (*FPs*) had significantly elevated 9-12 Hz power in sensor space.

Test-retest reliability of slow spindle events.

The MEG source estimates of slow spindle activity were excluded for two subjects. For one subject, the spindle detection threshold was >6 standard deviations above the mean. For the second subject, continuous high-amplitude 9-12Hz activity was detected for more than half of the nap which was deemed artifactual after visual inspection of the data.

Sensor space EEG slow spindle events were stable within individuals across two naps (ICC=.73, CI: [.27,.75]; Figure S7). Slow spindle events detected by only EEG showed a moderate ICC (EEG: ICC=.60, CI: [.27,.75]). Slow spindle event density using only MEG and combined EEG/MEG was not reliable across sessions (MEG: ICC=.35, CI: [.05,.67]; EEG/MEG: ICC=.47, CI: [.06,.76]).

Comparison of source space slow spindle events detected with EEG alone, MEG alone and combined EEG/MEG.

Overall slow spindle event density differed significantly between source estimates (*F*(2,71)=165.46,*p*<.001): Spindle event density was lower in MEG than either EEG (53%; *t*=8.34, *p*<.001) or EEG/MEG (49%; *t*=10.75, *p*<.001), and lower for EEG/MEG than EEG (7%, *t*=2.59, *p*=.02; Supplementary Figure 8a). Sixty-eight % of EEG-detected spindle events had no corresponding event in MEG. Conversely 32% of MEG-detected spindle events lacked a corresponding EEG event (Supplementary Figure 8b). Like fast spindles the combined EEG/MEG estimate captured more of the spindles present in EEG alone than MEG alone (*t*=15.24, *p*<.001). Similarly, there were more common spindles between the combined EEG/MEG and the MEG alone than between the EEG alone and MEG alone (*t*=5.44, *p*<.001).

The spatial distribution of slow spindle density differed qualitatively across modalities but was similar to fast spindle topography. EEG showed maximum spindle density in lateral and medial frontal cortex, while MEG spindle density was relatively low over prefrontal cortex and peaked in sensory and somatosensory areas (Supplementary Figure 3d-f).

Spindle events detected by EEG included more regions than MEG (*t*=12.50, *p*<.001) or combined EEG/MEG (*t*=11.52, *p*<.001). EEG/MEG detected spindle events were more widespread than those detected by MEG (*t*=12.91, *p*<.001; Supplementary Figure 8c). There were topographical differences between modalities: MEG was less likely than EEG to detect medial and lateral frontal spindle events (Supplementary Figure 9a) and EEG was less likely than MEG to detect spindle events in motor and somatosensory cortex (Supplementary Figure 9b).

| **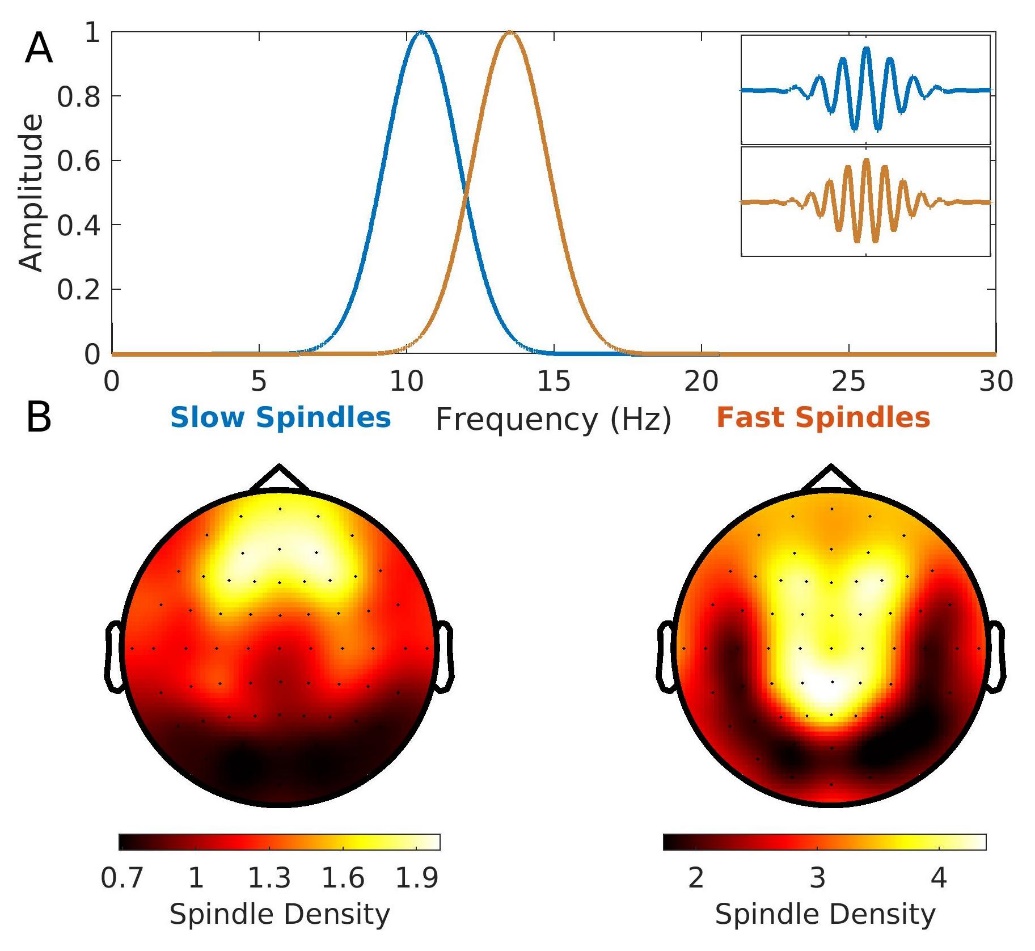** |
| --- |
| **Supplementary Figure 1**. A) Slow and fast spindles were detected using complex Morlet wavelets centered at 10.5 Hz (blue) and 13.5Hz (orange) respectively with a bandwidth of 3Hz. The wavelet bandwidth was defined based on the full width half max of the wavelet amplitude response in the frequency domain. Insets show the real part of each wavelet in the time domain (x-axis: 1 sec). B) Topography of EEG spindle density. As expected, slow spindles in sensor EEG show maximum density over frontal electrodes whereas fast spindle density shows maximum at central and parietal electrodes. |

| **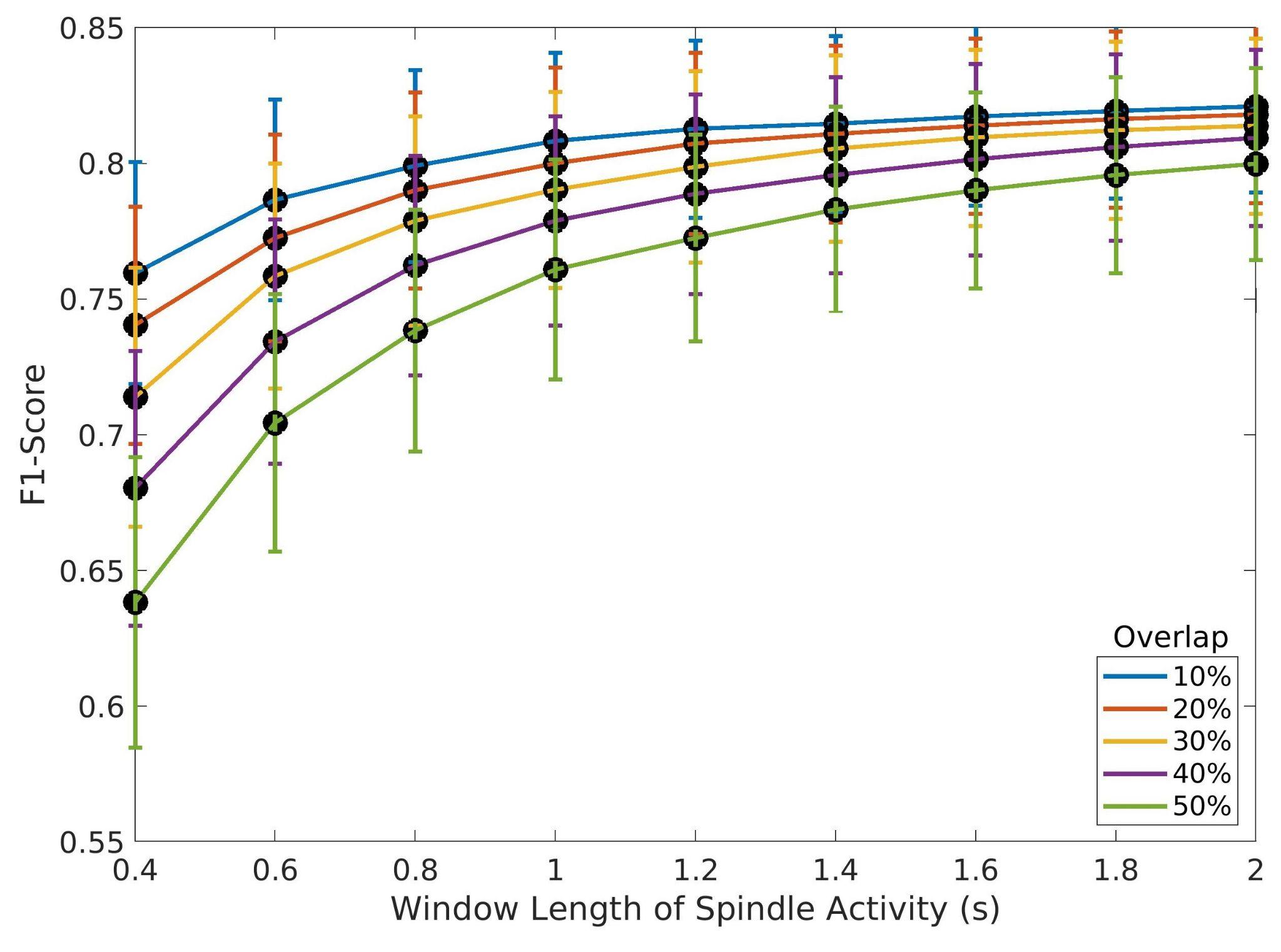** |
| --- |
| **Supplementary Figure 2.** Correspondence of fast spindle events (12-15 Hz) detected in sensor vs. source space EEG. *F1*-score plotted for different window lengths (.4 - 2 s with.2 s intervals) and overlap (10-50%). For our main analysis we used a window of 1 s length and a minimum overlap of 20%. |

| **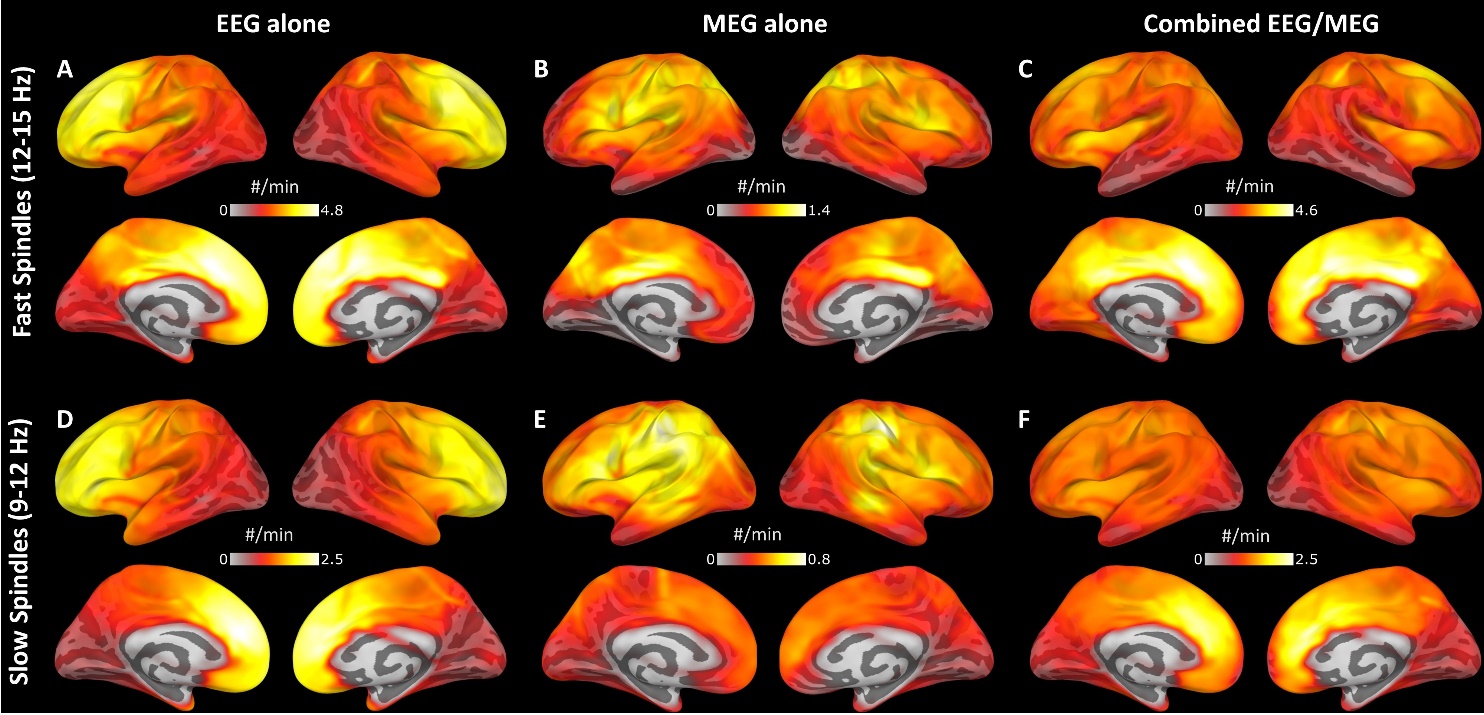** |
| --- |
| **Supplementary Figure 3.** Spatial distribution of fast spindle density in source space for A) EEG alone, B) MEG alone and C) combined EEG/MEG. D, E and F) Similar for slow spindle density. |

| **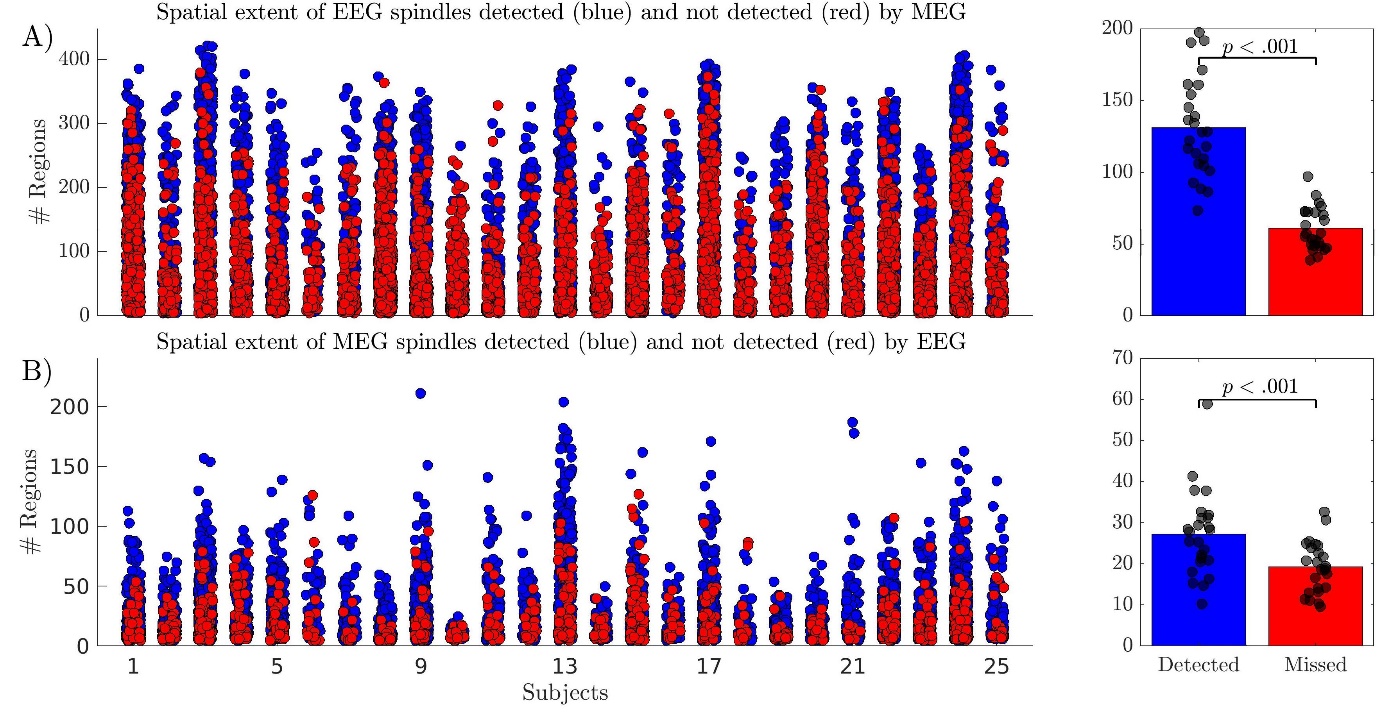** |
| --- |
| **Supplementary Figure 4.** Spatial extent of A) EEG fast spindle events detected (blue) and not detected by MEG (red), B) MEG fast spindle events detected (blue) and not detected by EEG (red). Spatial extent of detected and missed spindle events for each subject (left; each blue and red circle represent a spindle event) and averaged across subjects (right; black circle represent data for each subject). Both modalities tended to miss spindle events that were detected in fewer regions. |

| 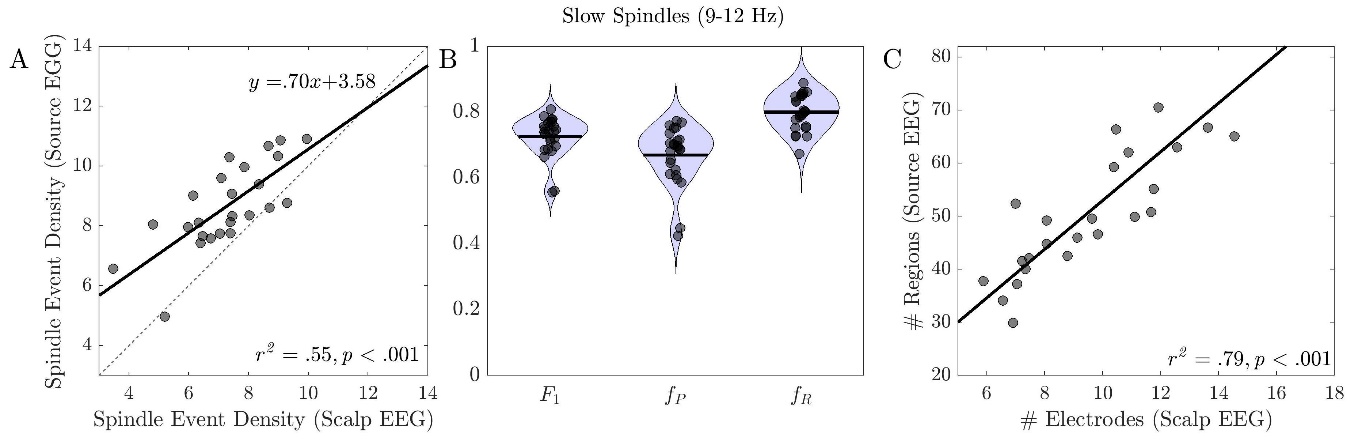 |
| --- |
| **Supplementary Figure 5.** Fast spindle events in source vs. sensor space EEG. A) Spindle event density in source vs. sensor space. Regression line (black solid) and the identity line (grey dashed) are shown. B) Correspondence of spindle events detected at the source vs. the sensor space (F1 = .73±.06, *f*_P_= .67±.09, *f*_R_=.80±.05). C) Spatial extent of spindle events in source vs. sensor space with regression line. |

| 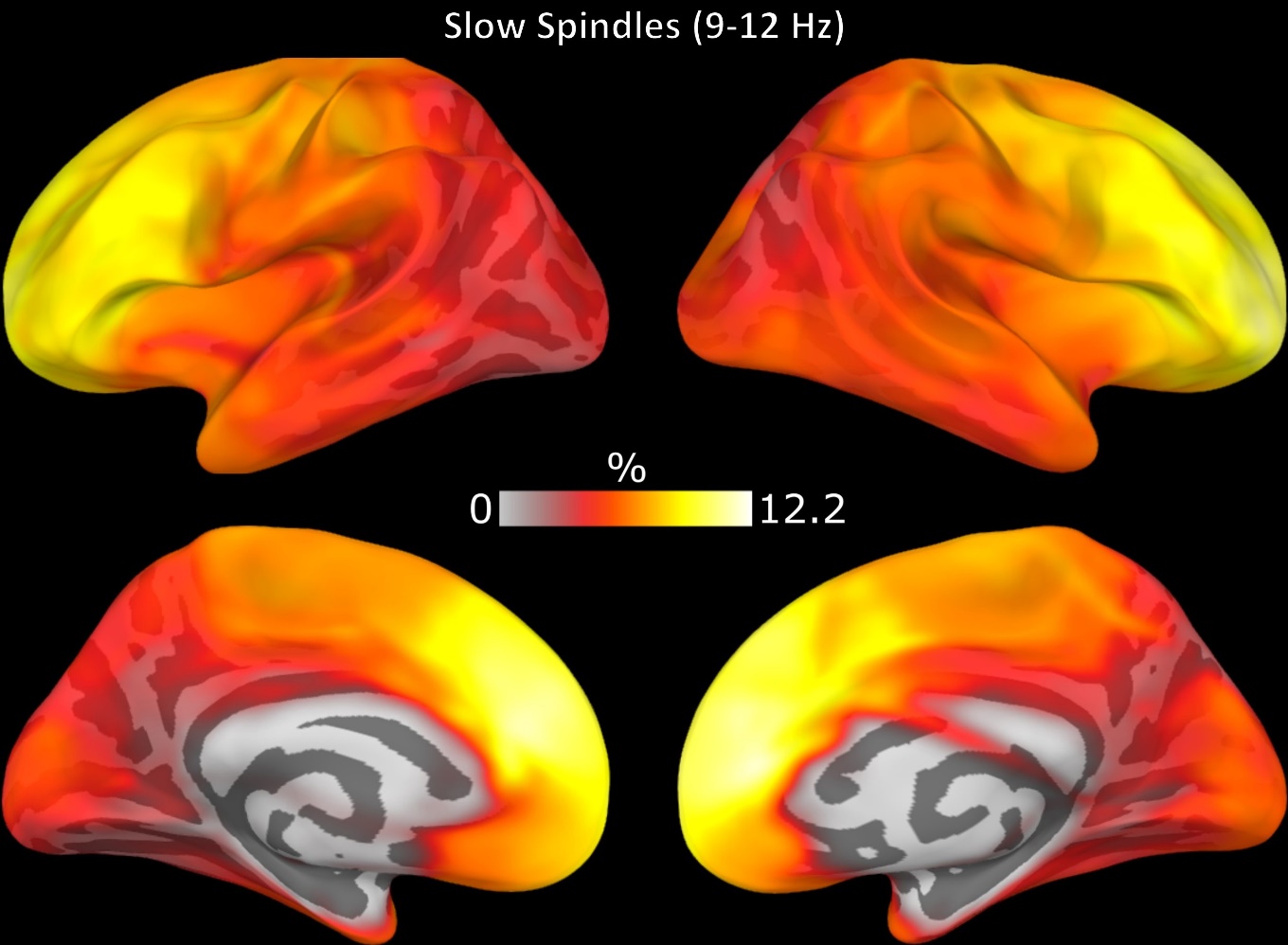 |
| --- |
| **Supplementary Figure 6.** Topography of slow spindle events detected only in source space EEG (FPs). The color of each region represents the number of *FPs* expressed in this region over the total number of *FPs* as a percentage. |

| 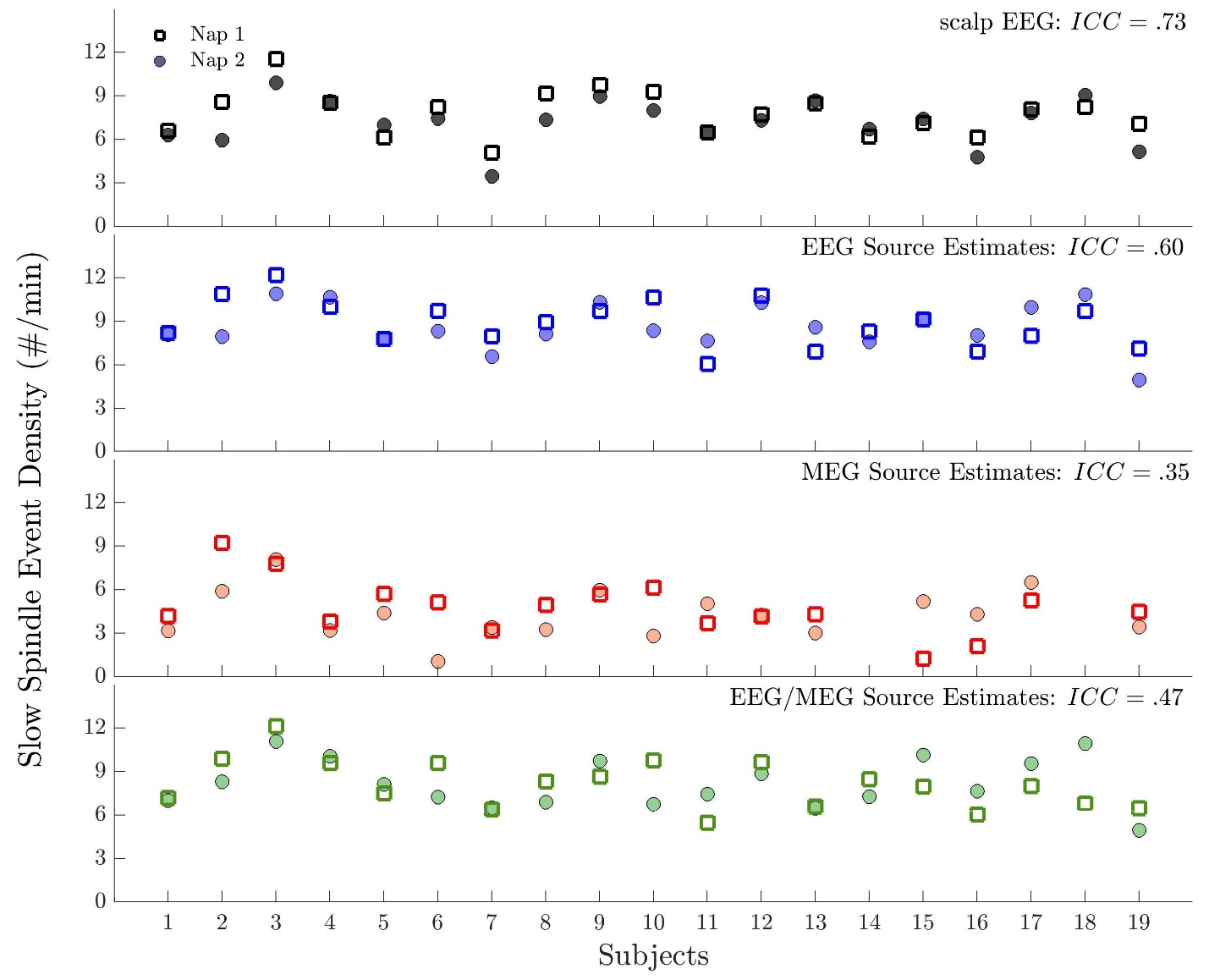 |
| --- |
| **Supplementary Figure 7.** Test-retest reliability of slow spindle events across naps for each modality. Plot of spindle event density for each subject during Nap 1 and Nap 2. Spindle events were detected (from top to bottom) at scalp EEG, source EEG, MEG and EEG/MEG. Note that the MEG source estimates were excluded for two subjects (see Supplementary Results). |

| 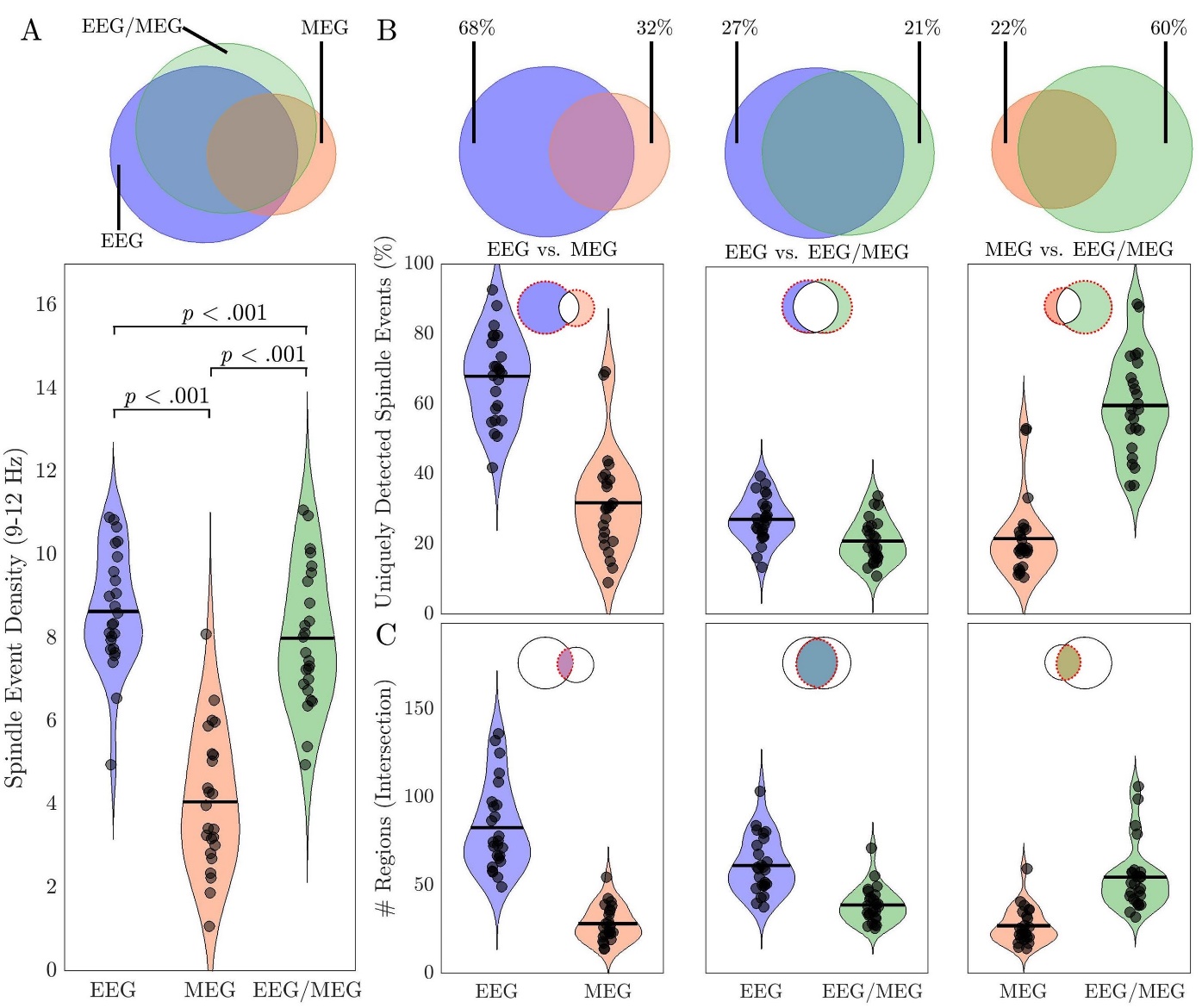 |
| --- |
| **Supplementary Figure 8.** Slow spindle events in source space using EEG alone, MEG alone and combined EEG/MEG. A) Venn diagram and violin plots depicting spindle event density in EEG, MEG and EEG/MEG with p-values for pairwise comparisons. B) Percent of uniquely detected spindle events by each modality for EEG vs. MEG, EEG vs. EEG/MEG and MEG vs EEG/MEG. C) Spatial specificity of commonly detected spindle events (intersection of Venn diagrams). Spatial extent of spindle events detected by EEG and MEG, EEG and EEG/MEG, and MEG and EEG/MEG. Black circles represent individual data. |

| 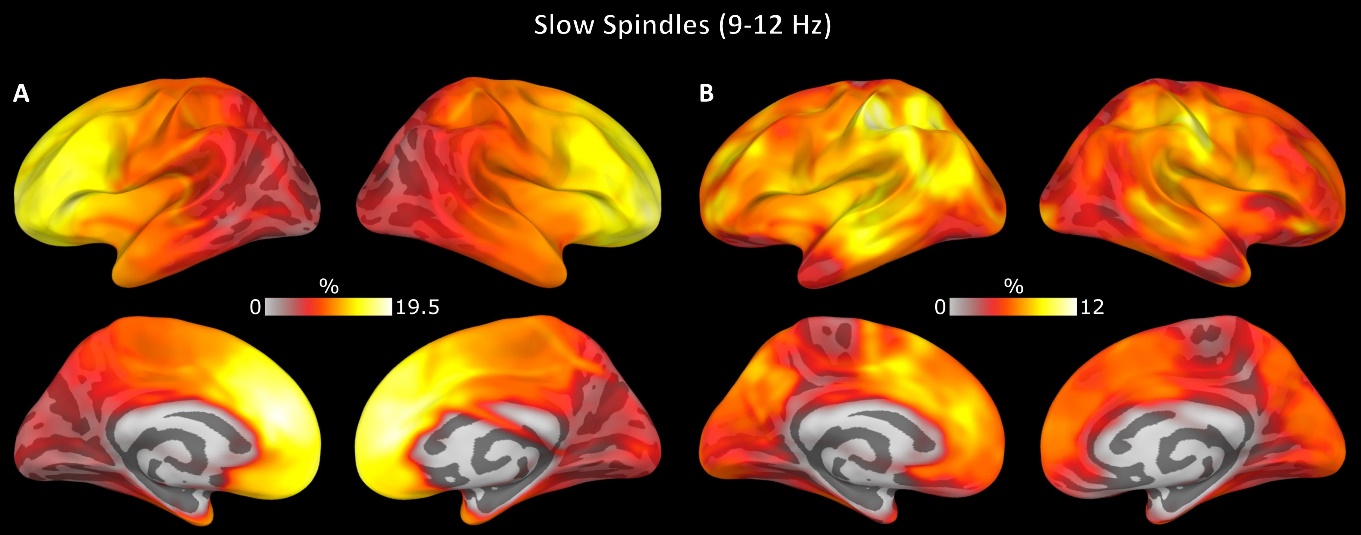 |
| --- |
| **Supplementary Figure 9.** Topography of spindle events uniquely detected by A) EEG and B) MEG. The color represents the percent of spindle events detected in each region relative to the total spindle events detected. |
